# A Case Report of Herpes Simplex-1 Viral Encephalitis Complicated by Hemorrhagic Conversion

**DOI:** 10.7759/cureus.24255

**Published:** 2022-04-18

**Authors:** Jane Ehret, Ali Al Safi, Chika Akabusi, Oluwafemi Ajibola, David Kung

**Affiliations:** 1 Internal Medicine, Nuvance Health at Vassar Brothers Medical Center, Poughkeepsie, USA; 2 Critical Care Medicine, Nuvance Health at Vassar Brothers Medical Center, Poughkeepsie, USA

**Keywords:** viral meningoencephalitis, infectious encephalitis, intracranial hemorrage, herpes simplex virus type 1, hemorrhagic conversion, hsv encephalitis

## Abstract

Herpes simplex virus (HSV) encephalitis is the most common cause of nonendemic sporadic encephalitis in the United States. Treatment with acyclovir has been proven to reduce mortality by 50%. Antiviral therapy should be initiated immediately in patients with clinical suspicion of viral encephalitis and should not be delayed by serological confirmation of the diagnosis. The most common central nervous system complication of HSV encephalitis is seizures (38%), while intracranial hemorrhage is very rare (2.7%). We describe a case of a 59-year-old African American male who presented to the hospital after being found unresponsive for a day and was found to have HSV-1 encephalitis that was complicated by hemorrhagic conversion. Our patient's neurological status did not improve even with appropriate antiviral treatment with a 28-day course of intravenous (IV) acyclovir. Intracranial hemorrhage is a rare complication in patients with HSV encephalitis. Close monitoring of neurological status is recommended for signs of deterioration or lack of improvement, and further imagings are needed (as in our patient) to evaluate for neurological complications such as intracranial hemorrhage.

## Introduction

Encephalitis is the inflammation of the brain parenchyma with evidence of neurologic dysfunction. Although the etiology of encephalitis is unknown in most patients, viruses remain the most well-known cause of encephalitis [1]. Herpes simplex 1 encephalitis (HSV-1) is the most common cause of nonendemic sporadic encephalitis in the United States. Viral meningitis is more common with Herpes simplex 2 encephalitis (HSV-2) than with HSV-1. HSV-1 is associated with significant morbidity and mortality of up to 70% without treatment [1,2]. Treatment with acyclovir has been proven to reduce mortality to approximately 20% [3]. The most common central nervous system complication of HSV encephalitis is seizures (38%), while intracranial hemorrhage is very rare (2.7%) [4]. We report a case of HSV-1 encephalitis that was complicated by hemorrhagic conversion.

## Case presentation

A 59-year-old African American man with a past medical history of diabetes mellitus, hypertension, and deep vein thrombosis on apixaban presented to our hospital after being found unresponsive for 24 hours. Upon arrival, he had a temperature of 37.9 °C, a heart rate of 119 bpm, a respiratory rate of 28 bpm, and blood pressure of 144/85 mmHg. On examination, he was somnolent but arousable to verbal stimuli and had expressive aphasia. Initial labs showed neutrophilic leukocytosis (WBC of 10.1; neutrophil of 86.7%), mild hyponatremia of 134 mmol/L, hemoglobin A1C of 12.4% (Table 1), and urinalysis suggestive of urinary tract infection (UTI) (Table 2).

**Table 1:** Initial laboratory tests Laboratory values on admission were significant for mild leukocytosis, mild hyponatremia, a normal hemoglobin, and an elevated HbA1c.

Initial laboratory tests	Results	Normal values
White blood cell (WBC) count	10.1 x10^9^/L	4.5-11.0 × 10^9^/L
Hemoglobin	13.4 g/dL	13.5 to 17 g/dL
Platelet	241 x10^9^/L	150-400 × 10^9^/L
Hemoglobin A1c	12.4%	< 5.7%
Glucose	166	65 to 99 mg/dL
BUN	20.2	6 to 20 mg/dL
Creatinine	1.07	0.70 to 1.20 mg/dL
Sodium	134	136 to 145 mmol/L
Potassium	3.9	3.5 to 5.1 mmol/L
Chloride	99	98 to 107 mmol/L
CO2	23	23 to 29 mmol/L
Calcium	8.8	8.6 to 10 mg/dL
ALT	17	7 to 40 IU/L
AST	22	15 to 41 IU/L
ALP	85	38 to 126 IU/L
Total protein	7	6 to 8.3 gm/dL
Albumin	3.8	3.5 to 5,0 gm/dL
Globulin	3.2	2.0 to 4.5 gm/dL
Bilirubin total	1.8	0.3 to 1.2 mg/dL
Hemoglobin A1c	12.4%	< 5.7%

**Table 2:** Urinalysis findings mg/dL: milligram per deciliter, HPF: high power field

Urinalysis	Results	Normal values
Appearance	Yellow and clear	Yellow and clear
Specific gravity	1.029	1.005-1.030
Glucose	300 mg/dL	Negative
Ketones	20 mg/dL	Negative
Blood	0.1 mg/dL	Negative
Protein	50 mg/dL	Negative
Nitrate	Negative	Negative
Leukocyte esterase	500 Leu/uL	Negative
White blood cells (WBC)	21-50/HPF	3-5/HPF
Red blood cells (RBC)	3-5/HPF	0-2/HPF
Bacteria	Moderate/HPF	None

**Figure 1:**
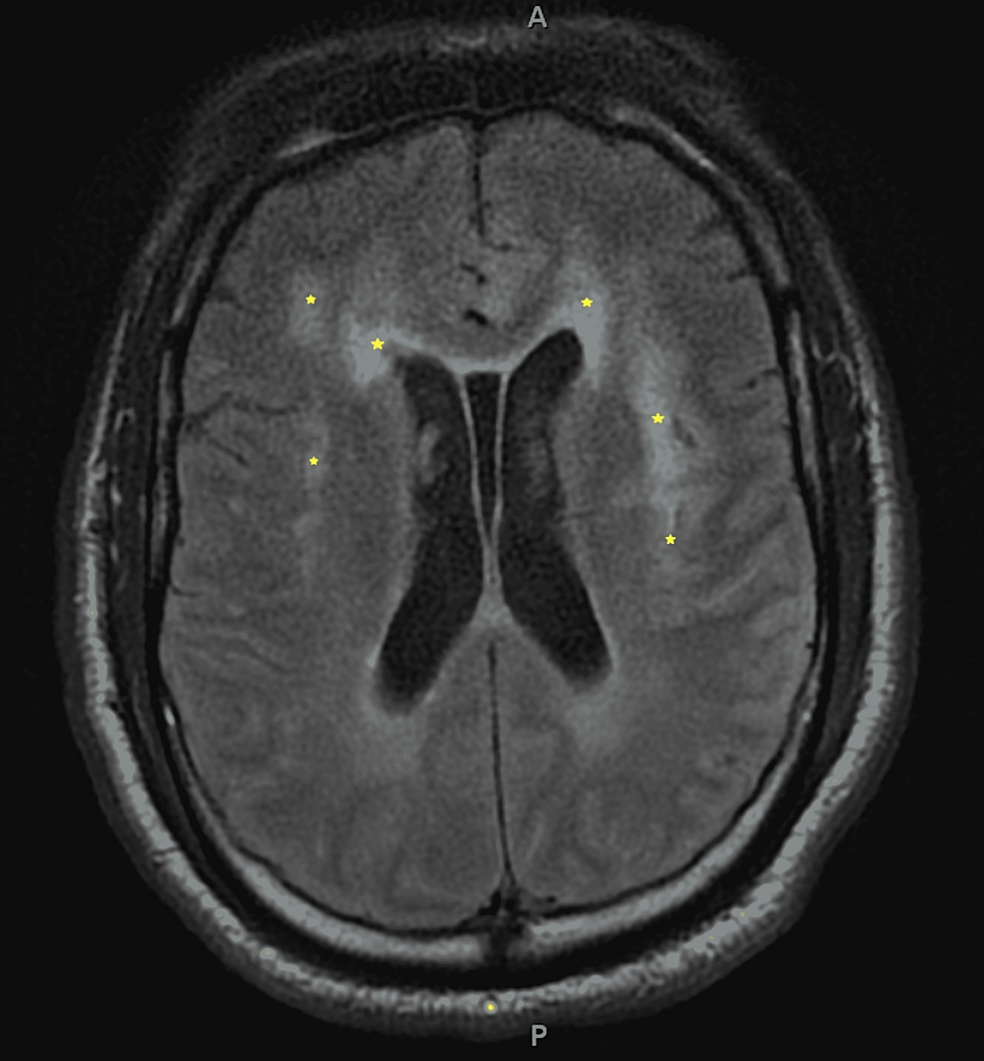
Axial T2 FLAIR MRI Brain prior to hemorrhagic conversion *Multiple areas of bilateral restricted diffusion involving the medial left anterior frontal lobe, left parietotemporal lobe and bilateral insula suspicious for encephalitis

The non-contrast head CT and CTA head/neck were unremarkable. The patient was started on IV ceftriaxone for UTI and admitted for sepsis and acute metabolic encephalopathy secondary to UTI. On hospital day 3, he became increasingly febrile, obtunded, and minimally responsive to painful stimuli. The patient was emergently intubated because his airway was compromised from his obtunded state. MRI of the brain showed multiple areas of bilateral restricted diffusion involving the medial left anterior frontal lobe, left parietotemporal lobe, and bilateral insula (worse on the left) suspicious for encephalitis (Figure 1).

Continuous EEG revealed focal areas of cortical irritability in the temporal, frontal, and parietal regions on the left side. Lumbar puncture was delayed, as the patient was on Apixaban. He was treated empirically for meningitis with ceftriaxone, vancomycin, ampicillin, acyclovir, and dexamethasone. On hospital day 7, the patient underwent a lumbar puncture that showed WBC of 9, RBC of 4, elevated glucose of 86, and protein of 174; CSF gram stain was negative. The CSF HSV-1 PCR returned positive, confirming the diagnosis of herpes encephalitis (Table 3).

**Table 3:** Cerebrospinal Fluid Analysis

Cerebrospinal fluid analysis	Results	Normal values
Appearance	Colorless and clear	Colorless and clear
White blood cells (WBC)	9 cells/mcL	0-5 cells/mcL
Red blood cells (RBC)	4 cells/mcL	Nil
Glucose	89 mg/dL	50 to 80 mg/dL
Protein	174 mg/dL	15 to 60 mg/dL
Herpes simplex virus 1 (HSV-1) polymerase chain reaction (PCR)	Detected	Negative
Acid fast bacilli culture	No growth	No growth
Gram stain and bacterial culture	No growth	No growth

**Figure 2:**
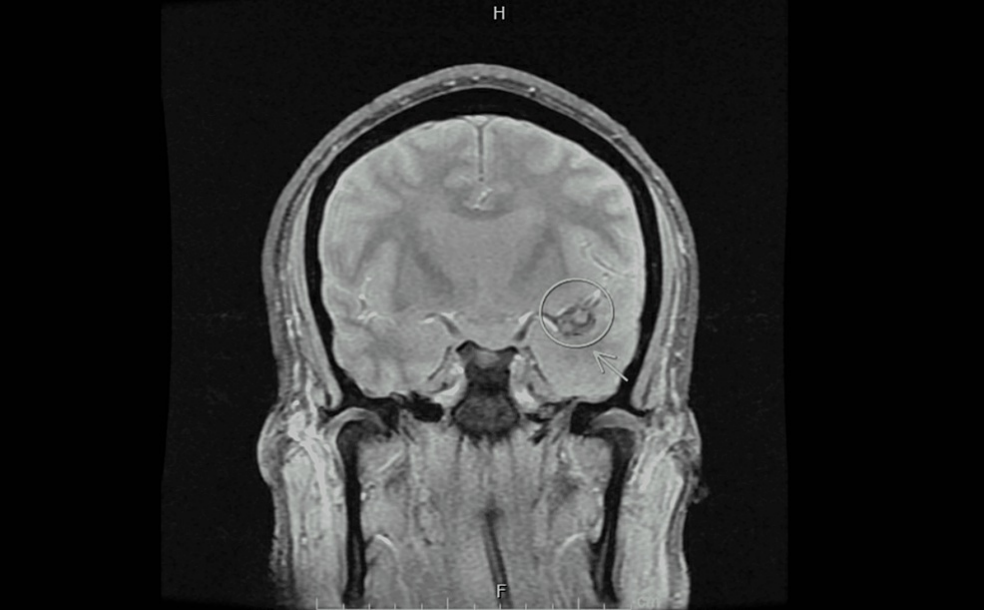
GRE T2-Weighted MRI Brain: Coronal view *Developing hypointense signal abnormality in medial left temporal lobe, suggesting an early hemorrhagic conversion of underlying encephalitis

Antibiotics were discontinued and he continued IV Acyclovir. The patient’s anticoagulation was resumed. Despite treatment, his mental status was not improving. On hospital day 13, a second brain MRI revealed a developing hemorrhagic conversion in the medial left temporal lobe (Figure 2). 

**Figure 3:**
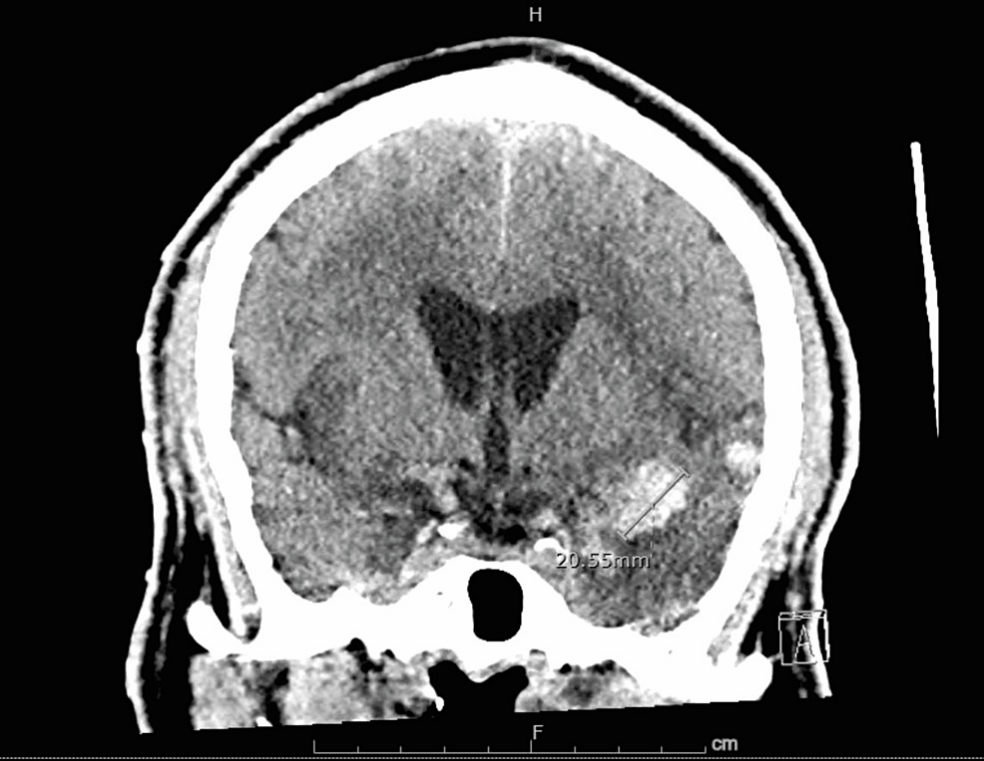
Non-contrast computed axial tomography of the head showing a hemorrhage in the left temporal lobe Hyperdensity measuring 3.6 x 1.6 x 2.3 cm along the anterosuperior margin of the temporal lobe.

**Figure 4:**
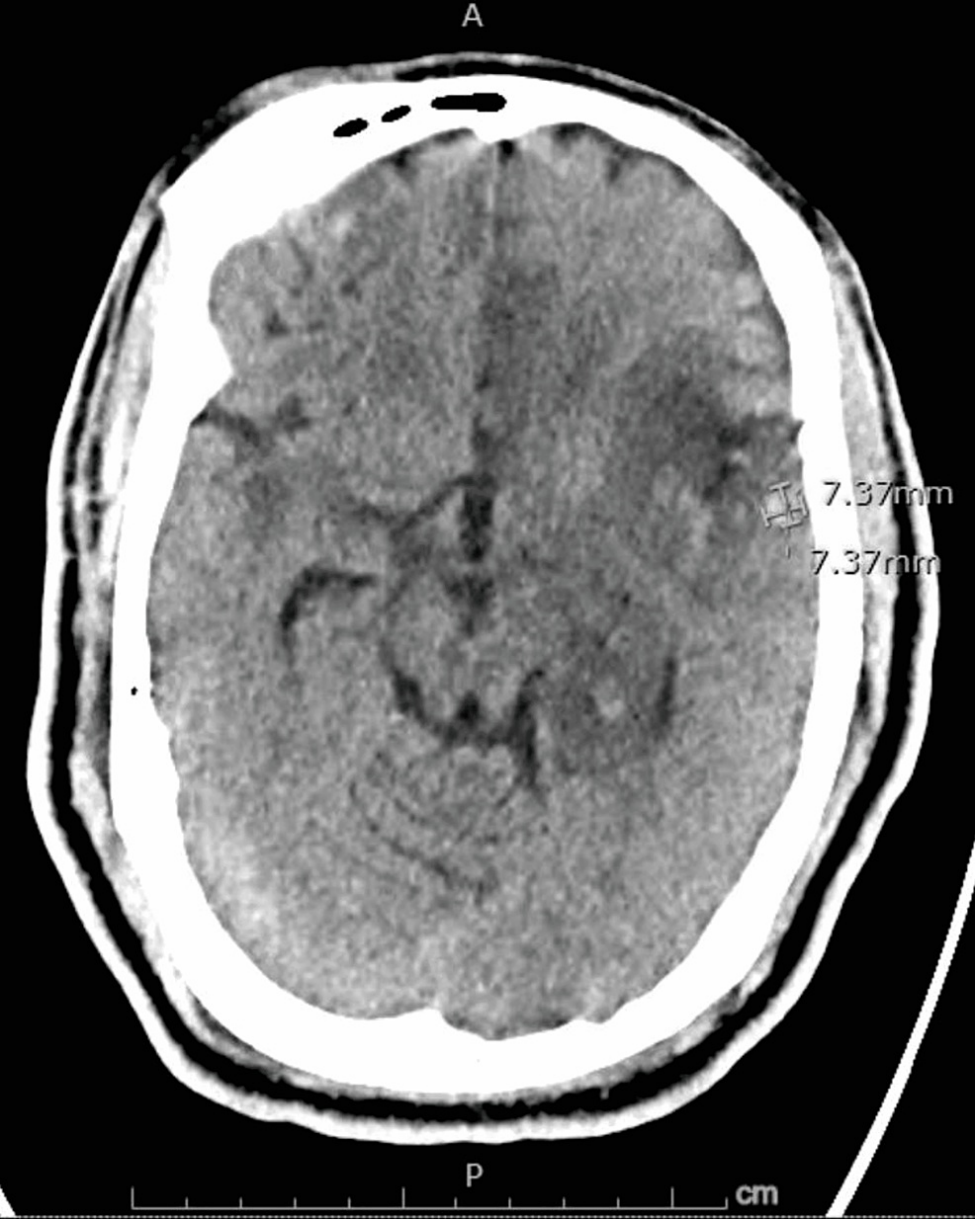
Non-contrast computed axial tomography scan of the head showing a hemorrhage, measuring 7.37 x 7.37 x 7 mm along the lateral aspect of the left temporal lobe.

**Figure 5:**
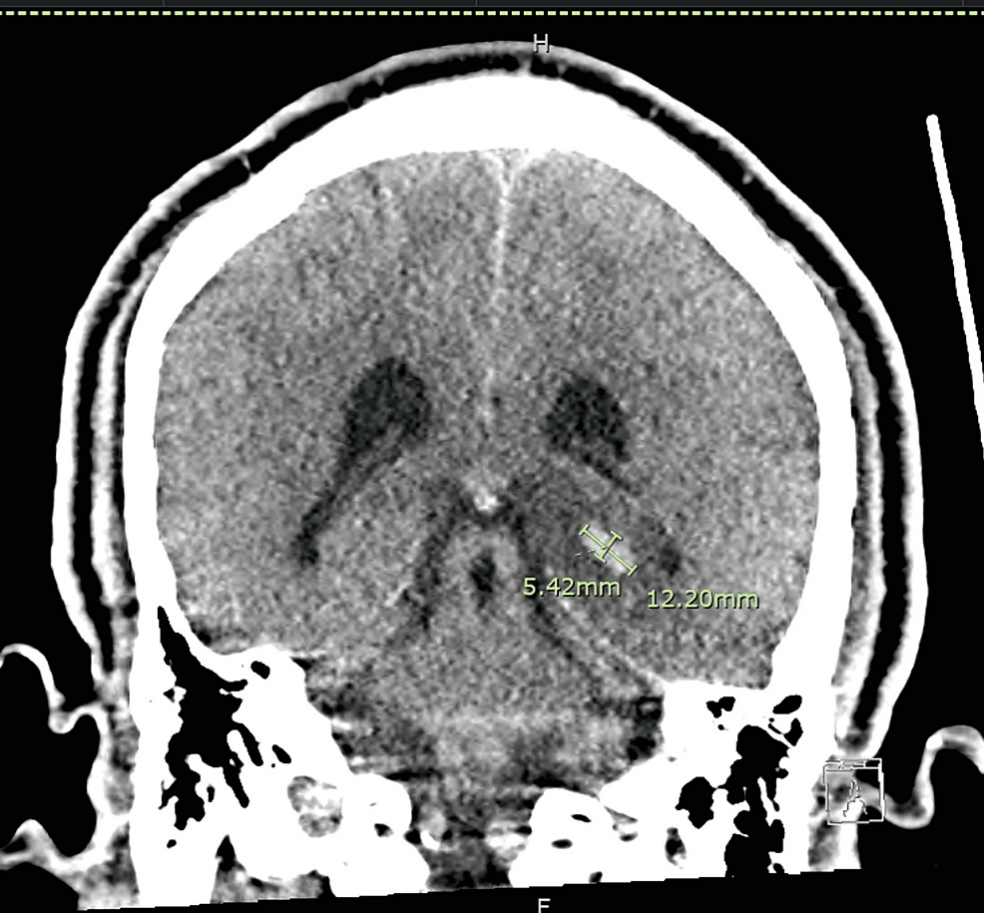
Non-contrast computed axial tomography of the head showing a hemorrhage, measuring 5 x 12 x 10 mm along the posterior medial aspect of the left temporal lobe.

Two days later, a noncontract head CT demonstrated increasing hemorrhages in the left temporal lobe, measuring up to 3.6 x 1.6 x 2.3 cm along the anterosuperior margin of the temporal lobe, 7 x 7 x 7 mm along the lateral aspect of the left temporal lobe, and 5 x 12 x 10 mm along the posterior medial aspect of the left temporal lobe without a midline shift (Figures 3-5).

Systemic anticoagulation was discontinued. Surgical evacuation was deemed not appropriate. The patient completed a 28-day course of IV Acyclovir. The patient failed to wean off the ventilator, for which a tracheostomy was placed. Unfortunately, his mental status did not fully recover. He was ultimately discharged to a long-term care facility after 40 days of hospitalization. 

## Discussion

HSV 1 is the most predominant virus identified with the cases of intracerebral hemorrhage, as seen in our patient [3]. On imaging, necrotizing infiltrate with scattered foci of small hemorrhage in the brain is usually seen; but frank hematoma is rare and usually occurs during the second week after initial presentation [5-9]. Our patient’s MRI showed developing hemorrhagic conversion in the medial left temporal lobe.

The exact mechanism for hemorrhage is unclear. The hemorrhage was proposed to be likely due to small vessel vasculitis resulting in endothelial damage with secondary bleeding, which was explained based on the fibrinoid necrosis in the pathological samples of evacuated hematoma [5, 10-13]. Some theories stated that bleeding can be due to rupture and transient hypertension caused by raised intracranial pressure [9]. Another hypothesis includes an immune-mediated inflammatory reaction that would damage the brain tissue and make it susceptible to bleeding [13]. Our patient had no evidence of coagulopathy with normal platelet count and coagulation profile, but he was on apixaban for deep vein thrombosis of unknown duration. Although he was on an anticoagulant, a work-up for other causes was unremarkable for aneurysms or any vascular abnormalities on CTA. In addition, previous studies have not reviewed any association between intracranial hemorrhage and anticoagulation use in patients with HSV encephalitis. The hematoma was localized to the temporal area, which is typical for HSV. The occurrence of the hemorrhage at the site of an abnormal MRI signal suggests that changes in the brain parenchyma and vessels induced by the encephalitis carry a potential risk of spontaneous bleeding [10], so close monitoring of neurological status is recommended in these patients.

HSV-1 encephalitis can occur in all age groups and all seasons and can present with altered mental status, seizures, or focal neurological deficit as seen in our patient who presented with altered mental status and aphasia. The investigation for encephalitis includes lumbar puncture, MRI, EEG, and in a specific clinical context, testing for autoimmune etiologies. Lymphocytic pleocytosis can be seen in the CSF and PCR HSV is 95% sensitive and specific at 48 to 72 hours. Without treatment, the mortality from HSV encephalitis can be as high as 70% [1-3]. The lumbar puncture was delayed in our patient as he was on apixaban for DVT which needs a washout period after discontinuation for lumbar puncture [3,4]. Treatment with acyclovir has been proven to reduce mortality to approximately 20% [5]. Antiviral therapy should be initiated in patients with clinical suspicion of viral encephalitis. Serological testing should not delay treatment. Neuroimaging can support the diagnosis by demonstration of temporal lobe edema or hematoma by magnetic resonance image as well as spike and slow-wave activity on electroencephalogram [6-8]. MRI can help with earlier detection of HSV encephalitis compared to CT scan, and 90% of MRIs done within the first 2 days in patients with HSV encephalitis were abnormal, especially at the cingulate gyrus and medial temporal lobe [1, 5, 8]. The initial MRI and EEG in our patient were suggestive of HSV encephalitis. The central nervous system complications from HSV encephalitis include seizures, ischemic stroke, and intracranial hemorrhage. While seizures are the most common complication (38%), intracranial hemorrhage is very rare (2.7%) [4]. 

Unfortunately, despite treatment, our patient’s neurological status did not improve, necessitating tracheostomy and PEG tube placement, and was ultimately discharged to a long-term care facility.

## Conclusions

In patients with HSV encephalitis, intracranial hemorrhage is a rare complication and patients with HSV encephalitis should be monitored closely. Deterioration or lack of improvement in neurological status, as with our patient, should necessitate further imaging for neurological complications like intracranial hemorrhage. 
